# A case series investigation of association between co-morbid psychiatric disorder and the improvement in body mass index among patients with anorexia nervosa and eating disorder not otherwise specified of the anorexia nervosa type

**DOI:** 10.1186/s40337-015-0049-z

**Published:** 2015-03-26

**Authors:** Robin Goh

**Affiliations:** Department of Psychiatry, Singapore General Hospital, Outram Road, Singapore, 169608 Singapore

**Keywords:** Personality, Anorexia nervosa, Depression, Generalized anxiety disorder, Body mass index

## Abstract

**Background:**

Anorexia nervosa and eating disorder not otherwise specified* (not fulfilling Anorexia Nervosa DSM IV criteria) are increasing in Singapore. Patients with eating disorders may also present with other psychiatric disorders such as depression and anxiety. The paper aims to investigate the association of co-morbid psychiatric disorders with the improvement of body mass index (BMI) in these patients.

**Methods:**

A retrospective cohort analysis of 182 patients with anorexia and eating disorder not otherwise specified at a tertiary hospital was done. The clinical course of co-morbid psychiatric disorders was correlated with the improvement of body mass index.

**Results:**

109 patients were included in the analysis and the mean BMI on resolution of co-morbid psychiatric disorders was BMI 16.9. There is a significant association between the BMI groups and the resolution of co-morbid psychiatric disorders, *χ*2 = 10.2, p = .03. Patients in BMI group 5 (BMI 16.6 - 18.5) were noted to be significantly less likely to resolve their psychiatric co-morbidity compared to the other 4 groups. (OR = 0.323).

**Conclusion:**

Patients with anorexia nervosa and eating disorders not otherwise specified were at increased risk of having co-morbid psychiatric disorders and the clinical course of co-morbid psychiatric disorders appeared to correlate with improved BMI. Specifically patients with BMI < 16.5 with co-morbid psychiatric disorders were more likely to recover from their co-morbid psychiatric disorder with nutritional rehabilitation than patients with a higher BMI.

## Background

There are few studies found in the literature which correlate body mass index with psychiatric complications [1-3]. Depression is the most common psychiatric disorder to present concurrently with eating disorders [4-6]. The severity of the anorexia nervosa has been postulated to be linked with the severity of major depressive disorder [7].

Other psychiatric disorders commonly seen in patients with anorexia nervosa include anxiety and obsessive compulsive disorders. There are studies which indicated a change in personality with the degree of severity of anorexia nervosa [8]. Aspects of personality change may differ between individuals and can range from irritability, argumentativeness and obstinacy [9]. They are often highlighted by family member who noticed such changes as their eating disorder deteriorate. Medication appeared to be not efficacious in reducing the co-morbid psychiatric disorders such as depression, anxiety and obsessive-compulsive disorders [10-13] without effective nutritional rehabilitation.

The study will investigate the effect of nutritional rehabilitation (hence improving body mass index) and pharmacological treatment on patients with eating disorders and co-morbid psychiatric disorders.

## Methods

The study design was a retrospective cohort study of patients with eating disorders under the care of Department of Psychiatry, Singapore General Hospital from 01 January 2003 to 31 December 2010. Inclusion criteria included inpatients and outpatients with the age range of 12 years old to 40 years old fulfilling the DSM-IV criteria for anorexia nervosa and patients with the diagnosis of eating disorder not otherwise specified. (without fulfilling the criteria of amenorrhea i.e. absence of at least 3 menstrual cycles.) [14,15]. The age limit was set from 12 years old to 40 years old because patients less than 12 years old would be treated in a hospital specializing in pediatric care and would not be transferred to Singapore General Hospital. The upper limit of 40 years old was set because the epidemiology of first onset eating disorders usually occurred in the younger age group and the catchment for eating disorder patients above 40 years old was small. The period of study was limited from Jan 2003 to Dec 2010 because Singapore General Hospital Eating Disorder service was only set up in 2003. The cut off of Dec 2010 was an arbitrary cut off to facilitate completion of the study.

The selected patients must present with either anorexia nervosa or eating disorder not otherwise specified as well as a co-morbid psychiatric disorder. Co-morbid psychiatric disorders that were studied included that of major depressive disorder, generalized anxiety disorder, and obsessive-compulsive disorder as defined by Diagnostic and Statistical Manual for Mental Disorders-IV criteria.

The diagnosis of eating disorders and co-morbid psychiatric disorders were determined by the multi-disciplinary psychiatric team consisting of a psychiatric consultant, medical officers, and allied health professionals such as dietician and psychologists. Personality changes reported by family members such as irritability, being argumentative and obstinacy were also categorized under co-morbid psychiatric disorders.

The case notes of all eating disorder patients in the Department of Psychiatry, Singapore General Hospital were reviewed and those meeting the inclusion criteria would be selected. The reviewers would follow case note entries of the entire treatment duration of the selected patients. The body mass index recorded during documentation of improvements of the psychiatric co-morbidities (either by the treating physician or family members) would be used in the analysis.

Data mining was done by 2 independent doctors/reviewers from Apr 2012 to Mar 2013. Both doctors were trained together to ensure consistency in the data collection. 182 patients were selected based on the inclusion criteria.

The study was cleared by the Singhealth Centralised Institutional Review Board (CIRB) which operated in accordance with the International Conference on Harmonisation (ICH)/Singapore Guideline for Good Clinical Practices (SGGCP). The power of the study was calculated with the help of a statistician and was set at 80%.

### Statistical analysis

Statistical analysis was done using the Statistical Package for Social Sciences Version 21.0 (SPSS v21.0 for Windows XP). Parametric variables were analyzed using *T*-test while nonparametric variables were analyzed using Kruskal-Wallis, Man-Whitney U and Chi-square tests for continuous and categorical variables respectively. Statistical significance was set at 0.05. The data analyst was blinded to the data collection.

## Results

The 182 patients were divided into 5 groups based on the body mass index for a more detailed analysis. The upper limit of the body mass index was set at 18.5 while there were no restrictions on the lower limit. Group 1 consisted of patients with body mass index less than or equal to 10.5, Group 2 consisted of body mass index from 10.6 to 12.5, Group 3 consisted of body mass index from 12.6 to 14.5, Group 4 consisted of body mass index from 14.6 to 16.5 and lastly Group 5 consisted of body mass index from 16.6 to 18.5.

One hundred and eighty-two eating disorder patients had co-morbid psychiatric disorders and were initially selected for the analysis. Of these 182 only 109 patients had documented data on the resolution of their co-morbid psychiatric disorders. Patients who defaulted were assumed to have not recovered and were not included in the study. Of the 109, 87 fulfilled the anorexia nervosa criteria (DSM IV) while the remaining 22 fulfilled eating disorder not otherwise specified. (These patients will be classified under anorexia nervosa in the latest DSM-5.) Two patients fall under Group 1, 11 under Group 2, 42 under Group 3, 47 under group 4 and 7 under group 5.

Under Group 1, both patients had the co-morbid psychiatric disorder of major depressive disorder. 10 patients had depression in Group 2 of which one patient presented with both depression and obsessive compulsive disorder while another presented with depression and personality changes. The last patient in Group 2 had obsessive compulsive disorder. 32 patients had depression in Group 3 of which one patient presented with both depression and obsessive compulsive disorder. Another 9 had personality changes and one anxiety disorder. There were 36 patients with depression in Group 4. (Two patients had depression and anxiety disorder while another had depression and obsessive compulsive disorder.) 11 patients presented with personality changes. There were 5 patients with depression in Group 5 (of which 2 had depression and anxiety disorder). The remaining 2 patients had obsessive compulsive disorder and personality changes respectively.

Out of the 109 patients, 85 had major depressive disorder, 22 had personality changes, 5 had anxiety disorder and 5 had obsessive compulsive disorder. (Some patients had dual pathologies.)

Pharmacological treatment and nutritional rehabilitation were started for these patients and the body mass index was closely monitored until full resolution of the co-morbid psychiatric disorders. The mean BMI on resolution of comorbid psychiatric disorders was BMI 16.9 for these 109 patients.

Some patients with eating disorders had a chronic course and their co-morbid psychiatric disorders would not improve even with improvement of the body mass index. There were patients who defaulted follow ups and their data would be incomplete. The remaining 73 patients in the sample fell into this category, and their data would be analysed using the worst case scenario, i.e. their co-morbid psychiatric disorders did not improve with time.

**Figure 1:**
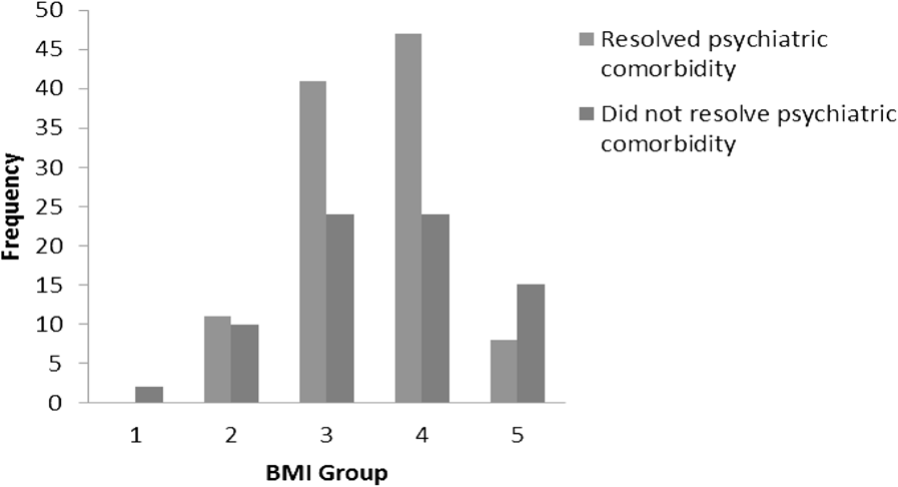
**Frequency of patients in the individual BMI groups who achieved resolution of their psychiatric comorbidities and patients who did not.** Only patients who developed a psychiatric comorbidity were involved. Legend: 1 BMI < 10.5, 2 BMI 10.6-12.5, 3 BMI 12.6-14.5, 4 BMI 14.6-16.5, 5 BMI 16.6-18.5.

The analysis was done using the Chi-square test. The frequency distribution of patients with psychiatric comorbidities was illustrated in Figure 1. This analysis attempted to correlate the BMI groups with the resolution of co-morbid psychiatric disorders. I.e. whether being in a particular BMI group would put you at a higher or lower risk of complete recovery from the co-morbid psychiatric disorders. All 182 patients with co-morbid psychiatric disorders on initial presentation were analysed by splitting them into their BMI groups and then comparing those with resolution of their co-morbid psychiatric disorders against those who had no resolution of their co-morbid psychiatric disorders.

The results demonstrated a significant association between the BMI groups and the resolution of co-morbid psychiatric disorders, *χ*^2^ = 10.2, *p* = .03. Specifically, patients in BMI group 5 (BMI 16.6 – 18.5), they were significantly less likely to resolve their psychiatric comorbidity compared to the other 4 groups. (OR = 0.323) There was no significant association for all other BMI groups (BMI groups 1, 2, 3, 4) with the resolution of co-morbid psychiatric disorders.

## Discussion

There have been studies which showed resolution of co-morbid psychiatric disorders with nutritional rehabilitations [16,17]. Based on the 109 complete data of eating disorder patients with co-morbid psychiatric disorders, the mean BMI on resolution of co-morbid psychiatric disorder was BMI 16.90. This arbitrary BMI 16.90 could be used as a gauge for determining resolution of the co-morbid psychiatric disorders. However, the confounding effect of treatment with medications should not be neglected and there is room for more research into comparing medications with nutritional rehabilitation.

In the second part of the analysis, BMI groups were compared to see if there were specific BMI groups which would influence the outcome of resolution of co-morbid psychiatric disorders. Patients in BMI group 5 (BMI 16.6 – 18.5) were significantly less likely to resolve their psychiatric co-morbidity compared to the other 4 groups. *χ*^2^ = 10.2, *p* = 0.03, OR = 0.323. This could be a result of the small sample size of only 7 patients. There was no significant association for all other BMI groups (BMI groups 1, 2, 3, 4) with the resolution of co-morbid psychiatric disorders.

The hypothesis from the results was that patients in BMI group 5 (BMI 16.6 – 18.5)’s co-morbid psychiatric disorders might not be secondary to their eating disorder. Hence the resolution of their eating disorder with the improvement of their BMI did not result in resolution of the co-morbid psychiatric disorder.

We have to be more cautious of co-morbid psychiatric disorders which were picked up during the initial consult for patients with BMI more than 16.6 since they are more unlikely to resolve them. They will need to be started on pharmacotherapy instead of just relying on nutritional rehabilitation to manage their conditions. (Nutritional rehabilitation on its own may help resolve co-morbid psychiatric disorders if they are secondary to the eating disorder).

### Limitations

This was retrospective analysis and it was difficult to draw a direct causal link between the low BMI and co-morbid psychiatric disorders. There may be also other potential confounders besides pharmacotherapy which may influence the outcome.

There was also limitation in the quality of the data collected as this was a retrospective study using an available eating disorder registry. There was no control over the completeness of data as well. For example, when we were trying to get the actual BMI during the resolution of the comorbid psychiatric disorder, we were unable to do so as the BMI might be recorded during an earlier consult instead of during complete recovery. We had to record the BMI based on the last observation carried forward. It would be ideal if BMI was directly recorded down onto the case notes during improvement of the co-morbid psychiatric disorders.

## Conclusion

Patients with anorexia nervosa and eating disorders not otherwise specified were at increased risks of having co-morbid psychiatric disorders. The clinical course of co-morbid psychiatric disorders appeared to correlate with the improvement of the body mass index. Specifically patients with BMI less than 16.5 with co-morbid psychiatric disorders were more likely to recover from their co-morbid psychiatric disorder with nutritional rehabilitation.
